# Adenomyoepithelioma of the breast coexisting with ductal carcinoma in situ: a case report and review of the literature

**DOI:** 10.1186/s40792-015-0083-8

**Published:** 2015-09-11

**Authors:** Mirei Kamei, Tsutomu Daa, Michiyo Miyawaki, Shuji Suehiro, Kenji Sugio

**Affiliations:** Department of Thoracic and Breast Surgery, Faculty of Medicine, Oita University, 1-1 Idaigaoka, Hasama, Yufu, Oita 879-5593 Japan; Department of Diagnostic Pathology, Faculty of Medicine, Oita University, 1-1 Idaigaoka, Hasama, Yufu, Oita 879-5593 Japan

**Keywords:** Adenomyoepithelioma of the breast, Ductal carcinoma in situ, Bloody nipple discharge

## Abstract

We herein report a case of adenomyoepithelioma (AME) of the breast with ductal carcinoma in situ (DCIS) involving a 71-year-old Japanese woman. She presented with bloody discharge from the left nipple. Mammography and ultrasonography showed a well-defined polygonal tumor. Fine-needle aspiration cytology of the mass and stamp cytology of the bloody nipple discharge showed malignancy. Mastectomy and a sentinel lymph node biopsy were performed. The final diagnosis was AME of the breast with DCIS. There are no reports of AME of the breast presenting with bloody nipple discharge; upon a diagnosis of AME of the breast with bloody nipple discharge, the possibility of the coexistence of breast cancer should thus be considered when encountering such cases.

## Background

Adenomyoepithelioma (AME) of the breast is a relatively rare benign neoplasm, first reported by Hamperl in 1970. It is characterized by the proliferation of epithelial and myoepithelial cells. AME of the breast occasionally occurs in the salivary gland and skin. Although AME of the breast is a benign neoplasm, there are three critical points in terms of the diagnosis and clinical course. The first point is the difficulty of diagnosis on fine-needle aspiration cytology, which often leads to a misdiagnosis of malignancy, according to the relevant literature. The second is that some reports have described recurrence and malignant degeneration. The third is that there are a few reports of AME of the breast coexisting with breast cancer. We herein report a case of AME of the breast coexisting with ductal carcinoma in situ (DCIS) diagnosed as malignancy at the preoperative stage.

## Case presentation

**Fig. 1:**
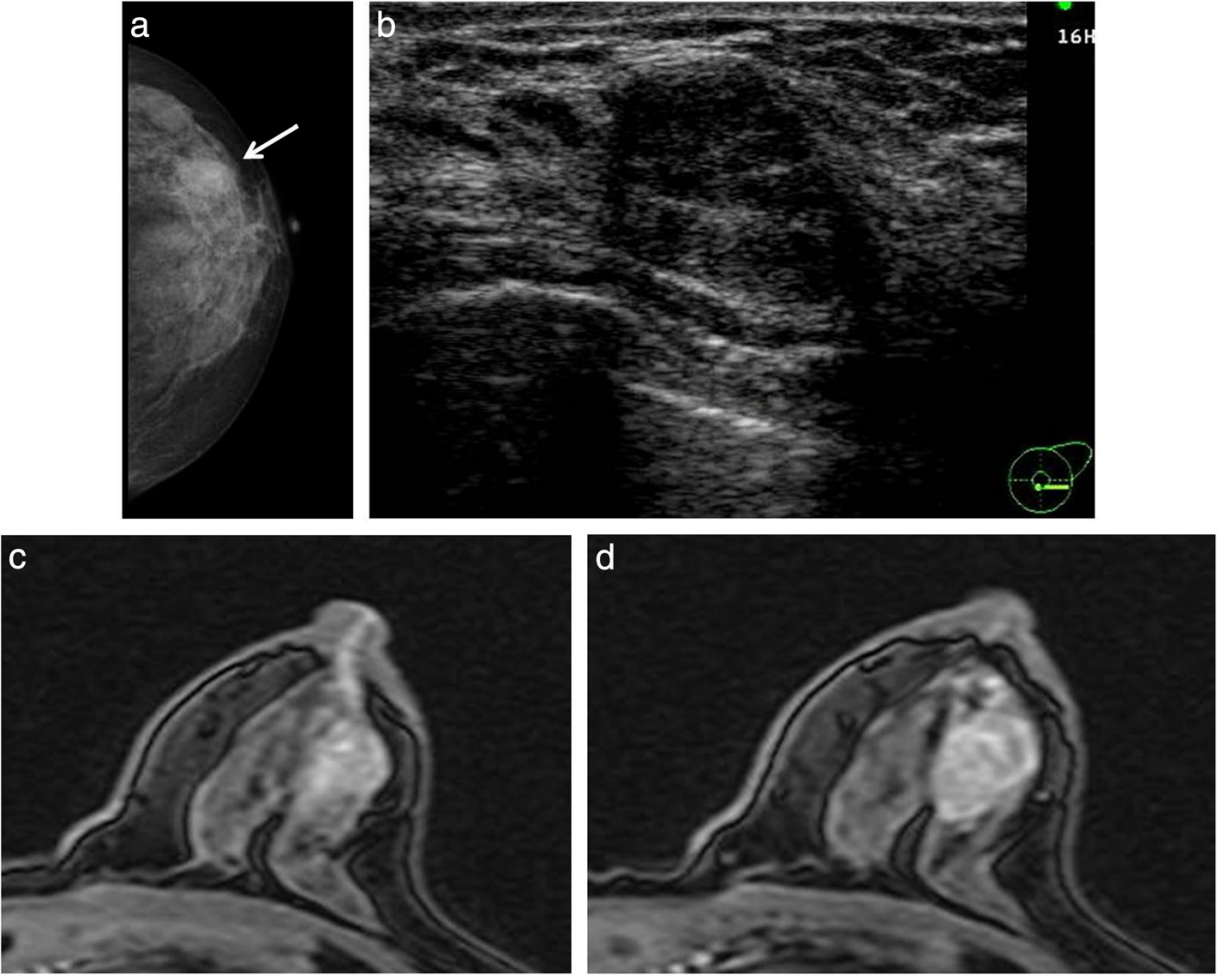
**a** Mammography showed an irregular oval mass (*arrow*) in the outer quadrant of the left breast. **b** Ultrasonography showed a well-circumscribed hypoechoic mass. **c** MRI showed high intensity from the mass to the nipple. **d** Enhanced MRI revealed a mass with an early peak and a delayed washout pattern and ring enhancement

**Fig. 2:**
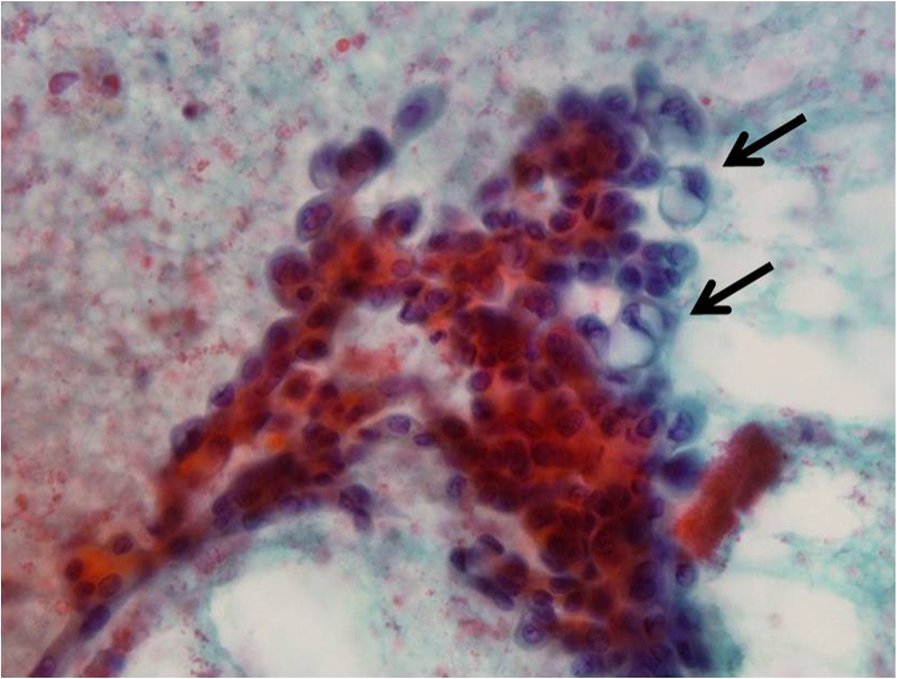
Stamp cytology of bloody nipple discharge revealed abundant clustered atypical cells and intracytoplasmic lumen (*arrows*)

**Fig. 3:**
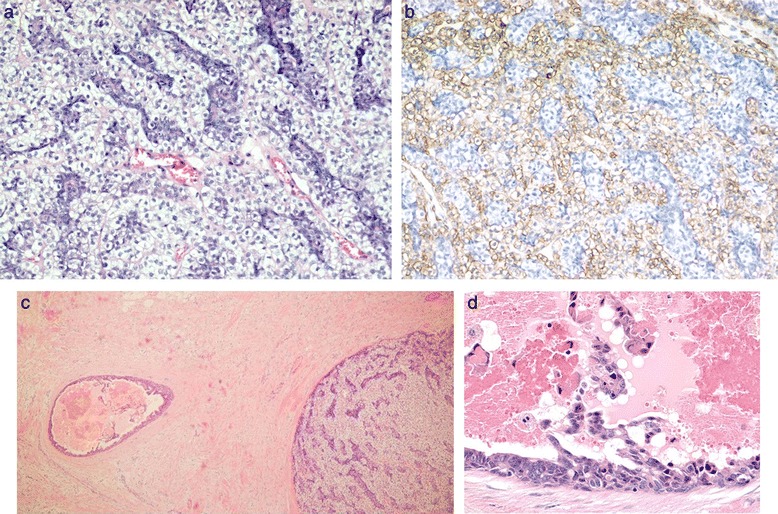
**a** The specimen showed proliferation of both epithelial and myoepithelial cells (HE staining, ×200). **b** Immunohistochemistry for SMA in the proliferating myoepithelial cells was positive (×200). **c** A surgical specimen showed ductal carcinoma on the side of the AME. **d** The epithelial cells of the dilated duct consisted of tumor cells, and there were many erythrocytes in the duct

A 71-year-old Japanese woman presented with bloody discharge from the left nipple. She had no medical or family history of breast disease. A physical examination showed a hard painless mass on the side of the nipple in the upper-outer quadrant of the left breast and bloody discharge from the left nipple upon compression. Mammography showed an ill-defined oval mass without microcalcification (Fig. 1a). Ultrasonography showed a well-circumscribed heterogeneous polygonal hypoechoic mass (Fig. 1b). Enhanced magnetic resonance imaging (MRI) of the breast revealed a lobulated mass measuring 19 × 14 × 13 mm in size with an early peak and a delayed washout pattern and rim enhancement (Fig. 1c, d). Fine-needle aspiration cytology of the mass revealed abundant clustered atypical cells, which showed an overlapping micropapillary structure. Individual cells had hyperchromatic nuclei, with a high nucleus-cytoplasm ratio, and clear nucleolus. The cytology of the bloody nipple discharge was similar to the findings of the mass and showed intracytoplasmic lumen (Fig. 2). Therefore, the tumor was diagnosed as a malignancy. Computed tomography showed neither enlarged lymph nodes nor distant metastasis. Mastectomy and a sentinel lymph node biopsy were performed. Microscopically, there were biphasic proliferations of myoepithelial cells and epithelial cells in the nodule lesion (Fig. 3a), and in portions of the lesion, myoepithelial cells were predominant. Although there were some mitotic and atypical cells, the mass was not malignant. According to immunohistochemistry, smooth muscle actin (SMA) and p63 were expressed in the myoepithelial cells (Fig. 3b), while AE1/AE3 was expressed in the epithelial cells. Therefore, the nodule was diagnosed as AME of the breast. There was a dilated mammary duct with necrosis on the side of the nodule (Fig. 3c). The epithelial cells of the dilated mammary duct with hyperchromatic nuclei showed low papillary proliferation (Fig. 3d). Therefore, the case was diagnosed as a low papillary-type ductal carcinoma in situ. The final pathological diagnosis was AME of the breast coexisting with DCIS. The patient did not undergo adjuvant therapy and is currently alive without recurrence 48 months after surgery.

### Discussion

Adenomyoepithelioma of the breast was first reported by Hamperl in 1970 [1]. It is a relatively rare benign neoplasm characterized by biphasic proliferations of myoepithelial cells and epithelial cells. In 1991, Tavassoli classified AME of the breast into three types: spindle type, tubular type, and lobulated type [2]. Typically, the discussion of AME of the breast involves the possibility of misdiagnosis of malignancy by a cytological analysis [3–5], and there are some reports of malignant transformation and recurrence [6]. Furthermore, AME of the breast may coexist with breast cancer. We herein report such a case, along with a review of the literature.

There have only been four reports of similar cases in the literature, and the chief complaint was a palpable mass [7–10] (Table 1). To the best of our knowledge, there have been no reports of a bloody discharge in AME. In our case, MRI showed a high signal from the mass to the nipple; thus, we initially believed that the mass had caused the symptom. However, the mass of AME was solid without necrosis, there were tumor cells among the epithelial cells in a dilated duct nearby, and there were many erythrocytes in the duct. Therefore, DCIS was thought to have been the cause of the bloody discharge. In our case, AME of the breast and DCIS were independent, providing no suggestion of the sequence of the derivation of one lesion from the other. One of the previously reported cases of DCIS were inside AME, while the other three cases showed DCIS adjacent to AME. Our case showed an early peak and washout pattern, suggesting invasive ductal carcinoma. In the literature, AME similarly showed a malignant pattern in MRI in two cases; thus, it appears to be difficult to distinguish between AME of the breast and invasive ductal carcinoma by MRI alone.

**Table 1:** Cases in the literature of adenomyoepithelioma with breast cancer

Author	Year	Age	CC	MMG finding	US finding	MRI	Cytology	Biopsy	Operation	Atypia	Within or out
Kuroda [7]	2008	66	Mass	Irregular lobulated mass	No record	No record	Suspicion of malignancy	IDC with neoplasm	Bp	−	Out
Han [8]	2010	55	Mass	Well-circumscribed mass	solid-cystic mass	Abnormal enhancement	–	AME with DCIS	Bp → Bt	+	Within
Warrier [9]	2013	55	Mass	WNL	Solid mass AS dilated duct	No record	Benign with atypia	AME with DCIS	Bp + SLNB Bt	+	Out
Maeda [10]	2013	35	Mass	FAD + Ca Calcification	Irregular mass Hypoechoic area	Rapid Gradual	–	IDC MP	Bt + SLNB	−	Out
Present case	2015	71	Bloody discharge	Well-circumscribed mass		Early peak washout	Malignancy	–	Bt + SLNB	+	Out

Within or out indicates ductal carcinoma inside or outside of AME

*CC* chief complaint, *MMG* mammography, *US* ultrasonography, *MRI* magnetic resonance imaging, *IDC* invasive ductal carcinoma, *Bp* partial mastectomy, *Bt* radical mastectomy, *WNL* within normal limits, *AS* acoustic shadow, *SLNB* sentinel lymph node biopsy, *FAD* focal asymmetric density, *MP* mastopathy

A review of the pertinent literature showed two cases with a suspected malignancy and one case which was difficult to diagnose according to fine-needle aspiration cytology. Although only one report described a diagnosis of AME of the breast by fine-needle aspiration cytology [11], in the previous literature, many cases of AME were operated according to a diagnosis of malignancy in cytology, despite the cases being indeterminate in imaging. In our case, mammography of AME showed an ill-defined mass lesion, while ultrasonography showed an oval hypoechoic mass, mimicking fibroadenoma. It was difficult to diagnose the patient clinically using only imaging. The reasons for the misdiagnosis of AME as carcinoma are thought to be due to the abundant variants of AME, the difficulty of detecting myoepithelial cells due to changes in morphology, and epithelial cells with atypia, not being considered in the differential diagnosis due to their rarity. However, the key feature in our case was that there was intracytoplasmic lumen only in the bloody nipple discharge. Therefore, a biopsy should be performed, as long as malignancy is not strongly suspected, if there is discordance between the imaging and cytological diagnoses.

For the treatment of AME, resection with an adequate margin is recommended to avoid recurrence. Among the previously reported cases, one case underwent partial mastectomy, and the others underwent mastectomy due to surgical margin positivity or being in the vicinity of a nipple. Lumpectomy may be adequate for AME only. However, there is a possibility of the coexistence of malignancy, as observed in the present case. If a diagnosis of AME is reached preoperatively, then we should carefully diagnose the patient according to imaging findings from different angles and develop a full account of the treatment plan. Our case was diagnosed as malignancy, and the tumor was near the areola; therefore, the patient underwent mastectomy. However, when diagnosis of AME with DCIS is made preoperatively, then microdochectomy should be considered.

## Conclusions

In cases of an AME diagnosis, we should keep in mind the possible coexistence of malignancy when making a differential diagnosis.

## Consent

Written informed consent was obtained from the patient for publication of this case report and any accompanying images. A copy of the written consent is available for review by the Editor-in-Chief of this journal.

## Abbreviations

AME: adenomyoepithelioma
DCIS: ductal carcinoma in situ
MRI: magnetic resonance imaging
SMA: smooth muscle actin
